# Cancer of the Stomach

**Published:** 1903-05-02

**Authors:** 


### CANCER OF THE STOMACH.

In an address delivered before the Leicester Medical Society Mr. Mayo Robson [1] made out a strong case for early operation in cases where cancer of the stomach is suspected. He shows that the results of partial gastrictomy for cancer are sufficiently encouraging in suitable cases to call for more general attention, while prolongation of life in comparative comfort may be assured by gastro-enterostomy in many of those cases where a removal of the growth is found to be impossible. He is also convinced that many deaths are ascribed to cancer when the disease has been inflammatory and curable by the operation of gastro-enterostomy. For satisfactory treatment early diagnosis is essential. The usual clinical signs are loss of appetite and wasting, with loss of strength coming on in a patient otherwise well. Or there may be listlessness and anæmia before any symptoms pointing to a stomach affection appear. Again, the attack may commence with pain after food and flatulent distention. Vomiting may be entirely absent, and is frequently not seen until a comparatively late stage. "Coffee ground" vomit may be seen, and occasionally pure blood may be brought up. Loss of flesh is well marked and is an early symptom. Pain is usual, but tenderness is absent unless there is some peritonitis. The most constant symptoms are pain, vomiting, and the presence of a palpable tumour. The presence of free hydrochloric acid is against a diagnosis of cancer, while the absence of hydrochloric acid is decidedly in favour of it. Rommelaere believes that the elimination of less than 180 grs. of urea per diem by a patient who suffers from chronic indigestion is pathognomonic of cancer; this is probably not absolutely true, but is suspicious. Mr. Robson thinks that it is permissible to assume that if a patient excretes as much as 450 grs. he is not suffering from carcinoma. If there is a palpable tumour present it may be very movable, whereas a tumour due to chronic ulcer of the pylorus is usually fixed under cover of the liver and ill defined. The presence of enlarged veins on the surface of the abdomen, of enlarged glands in the groin or over the left clavicle, or of other signs of secondary growths in the liver, bowels or peritoneum must all be taken into account in considering the case from a surgical standpoint. For the surgeon to obtain the best results the condition should be recognised before any lump can be felt, but the presence of a palpable tumour does not contra-indicate operation. In any case where a patient at or past middle age complains somewhat suddenly of indefinite gastric uneasiness, pain and vomiting, followed by progressive loss of weight and energy and associated with anæmia, cancer of the stomach should be suspected, and if no improvement takes place in a few weeks, exploratory operation is more than justified. Should this be done and the disease found to be cancer the subsequent procedure will depend upon (1) the position of the growth; (2) its extent; (3) the presence or absence of adhesions; (4) the presence or absence of secondary growths in the lymphatic glands or elsewhere. If the growth is at the cardiac end and involves the cardiac orifice, gastrostomy should be performed. If the growth is extensive, irremovable, and gastro-enterostomy is impossible, jejunostomy is indicated. Where the growth cannot be removed on account of adhesions, secondary growths, or extreme feebleness of the patient, gastro-enterostomy should be performed. By this means life is prolonged and made more comfortable, flesh and colour are regained, and the improvement in the condition of the patient may render a subsequent radical operation possible. In those cases where the disease is limited to the stomach, and where the lymphatic glands have not been seriously invaded, if the patient is in a sufficiently good condition, the radical operation should be done, and the growth entirely removed. Mr. Robson believes that the thorough removal of the disease may bring about as much relief to the patient as does the operation for the extirpation of cancer in the breast, and that in some cases a complete cure may follow.

[1] British Medical Journal, April 25th.

